# Visual perception of war images in Spanish TV news: an eye-tracking study using still frames

**DOI:** 10.3389/fnins.2025.1612487

**Published:** 2025-07-09

**Authors:** Miguel Ángel Martín-Pascual, Celia Andreu-Sánchez

**Affiliations:** ^1^Research and Development, Radio Televisión Española Insitute, Corporación RTVE, Barcelona, Spain; ^2^Neuro-Com Research Group, Institute of Neurosciences and Department of Audiovisual Communication and Advertising, Universitat Autònoma de Barcelona, Barcelona, Spain

**Keywords:** war images, visual perception, eye tracking, war journalism, visual fixations, visual memory, visual attention

## Abstract

Since Russia’s invasion of Ukraine in February 2022, screens across the world have been flooded with images of death and destruction in this territory in the West, as televisions globally have broadcast the war, bearing witness to the collapse of a community consumed by terror. This study aimed to investigate the visual perception of still frames of war images taken from Spanish TV news broadcasts. We showed participants (*N* = 49) a series of war images while tracking their gaze using an eye tracker and administered questionnaires related to the images. We analyzed how viewers allocated their gaze across three sections within the images—journalists, war imagery, and informative text—finding significant differences, with the longest gaze duration and highest number of fixations on war images, followed by the text section, and lastly, the journalists. We compared the time and the way viewers looked at war images featuring deceased individuals versus those without, finding significant differences, with more time and more fixations, but less revisits, dedicated to images containing deceased individuals. When comparing visual attention to leaders of the opposing war factions, Zelenski vs. Putin, the latter received more attention, with participants looking at Putin for a longer period, and this was also associated with stronger negative emotions. In assessing participants’ memory of the presented images, the majority of responses were correct, particularly regarding the content shown in the images, although some participants failed to recall certain elements; however, when it came to content not present in the images, most participants accurately avoided fabricating details that were not shown. These findings offer insights into how war is visually perceived in Spanish television news.

## 1 Introduction

The 2022 invasion of Ukraine by Russia brought a renewed flood of war imagery to television screens worldwide, depicting scenes of death and destruction far from the front lines (Ononiwu, 2024). Televisions worldwide have serialized the war—this time close to a society with Western values and daily life—witnessing the collapse of a community under terror (Webster and Neal, 2022; Eddy and Fletcher, 2022). The effect of these images on public opinion is easy to understand: we often lack elements for context and judgment, yet we experience an emotional upheaval when witnessing a society in ruins, although from an informational perspective, we cannot determine how an individual processes information, as this depends on subjective factors, context, proximity, and the person’s prior experiences (O’Loughlin, 2011). While the coverage of the Russian invasion of Ukraine has varied widely between Western and Russian media ecosystems, with propaganda, disinformation, and narrative bias present across both (Hanley et al., 2023), the use of frames has depended more on the goals of propaganda than on the actual events in the authoritarian Russian media system (Chernov, 2023). Public sentiment toward economic sanctions has also been heterogeneous (Ngo et al., 2022).

Covering the Russian war in Ukraine has been a challenge for the news media, in part, in deciding which images of war should be shown, the safety of journalists, or public engagement (Pavlik, 2023). Also, since war images are no longer only present in traditional media but also available on social media (Yarchi and Boxman-Shabtai, 2023), they are much more accessible for everyone. In this context, it seems interesting to investigate the impact of war images in the news on viewers. In this regard, several studies have analyzed images in times of war (Müller and Christ, 2023), while very few have used quantitative analysis techniques, such as eye tracking or psychophysical methods, except for assessing post-traumatic stress syndromes and emotional valence when viewing violence (Kimble et al., 2010; Ypsilanti et al., 2020). In this study, we used the eye-tracking technique that allows for a quantitative measurement of visual fixations, attention, and gaze behavior in areas of interest (Eckstein et al., 2017; Schütz et al., 2011; Tatler et al., 2010). The purpose of this research was to identify patterns of observation in subjects while viewing war images in the news.

## 2 The present study

In this research, we aimed to study the visual perception of war images in broadcast news. We asked five research questions: (1) When a journalist is broadcasting war information, which element captures more of the viewers’ gaze: the journalist, the war images, or the explanatory text? (2) Does an image depicting deceased individuals provoke a different visual perception compared with other war images? (3) Do the leaders of opposing war factions, such as Zelenski and Putin, generate distinct visual attention patterns in the audience? (4) And do they provoke different emotions in the audience? (5) Do audiences recall the content of war images? We expected that political leaders would elicit differential gaze patterns and emotional responses owing to their symbolic roles and differing portrayals in the media. Furthermore, we hypothesized that faces would receive more attention than text elements or images. Finally, we expected that participants would recall the content shown in the images more accurately rather than fabricating information that was not presented.

To investigate these questions, we planned an approach based on an eye-tracking study including self-reported data. We presented a set of war images to participants while registering their gaze with the help of an eye tracker, and we provided them with some questionnaires related to the images.

## 3 Materials and methods

### 3.1 Participants

Fifty participants were recruited for this experiment. Participants were recruited via university mailing lists and professional networks in Spain. The data collection took place over a period of 8 months, between May and December 2023. The participants were all based in Spain at the time of the study. Since this was a long period for data collection, media coverage may have influenced viewers’ perceptions and responses. This potential impact is discussed in the Limitations section of the manuscript. However, it is important to note that this study began more than a year after the onset of the invasion of Ukraine. By that time, participants had already been exposed to extensive media coverage, and the foundational narratives surrounding the conflict in Western media were well established. One participant was excluded owing to a poor calibration of the eye tracker (*N* = 49). A power analysis was conducted using G*Power 3.1 (Faul et al., 2009) to determine the appropriate sample size. Assuming a medium effect size (*d* = 0.5), a significance level of *α* = 0.05, and a desired statistical power of 0.95, this analysis indicated a required sample size of 47 participants. The final sample of 49 participants meets this criterion, supporting the sufficiency of the study’s statistical power. The mean (±SD) age of the participants was 34.755 (±16.586). Of the final 49 participants, 18 (36.7%) were men and 31 (63.3%) were women. Eight out of 49 were journalists (16.3%), while the rest (83.7%) had different professions. Only one indicated that they had covered a war conflict, while seven had previously informed about war. Aside from these, no participants reported first-hand experience with the war in Ukraine, proximity to the conflict zone, or the loss of someone due to the war. All carried out the test with normal vision, or vision corrected with glasses or contact lenses. The experiments followed the guidelines, procedures, and regulations for human research established by the Universitat Autònoma de Barcelona and received approval from its Ethics Commission for Research with Animals and Humans (CEEAH/CERec). This study complied with the principles of the Helsinki Declaration and other relevant international standards. All participants gave written informed consent before their participation.

### 3.2 Stimuli

**Figure 1:**
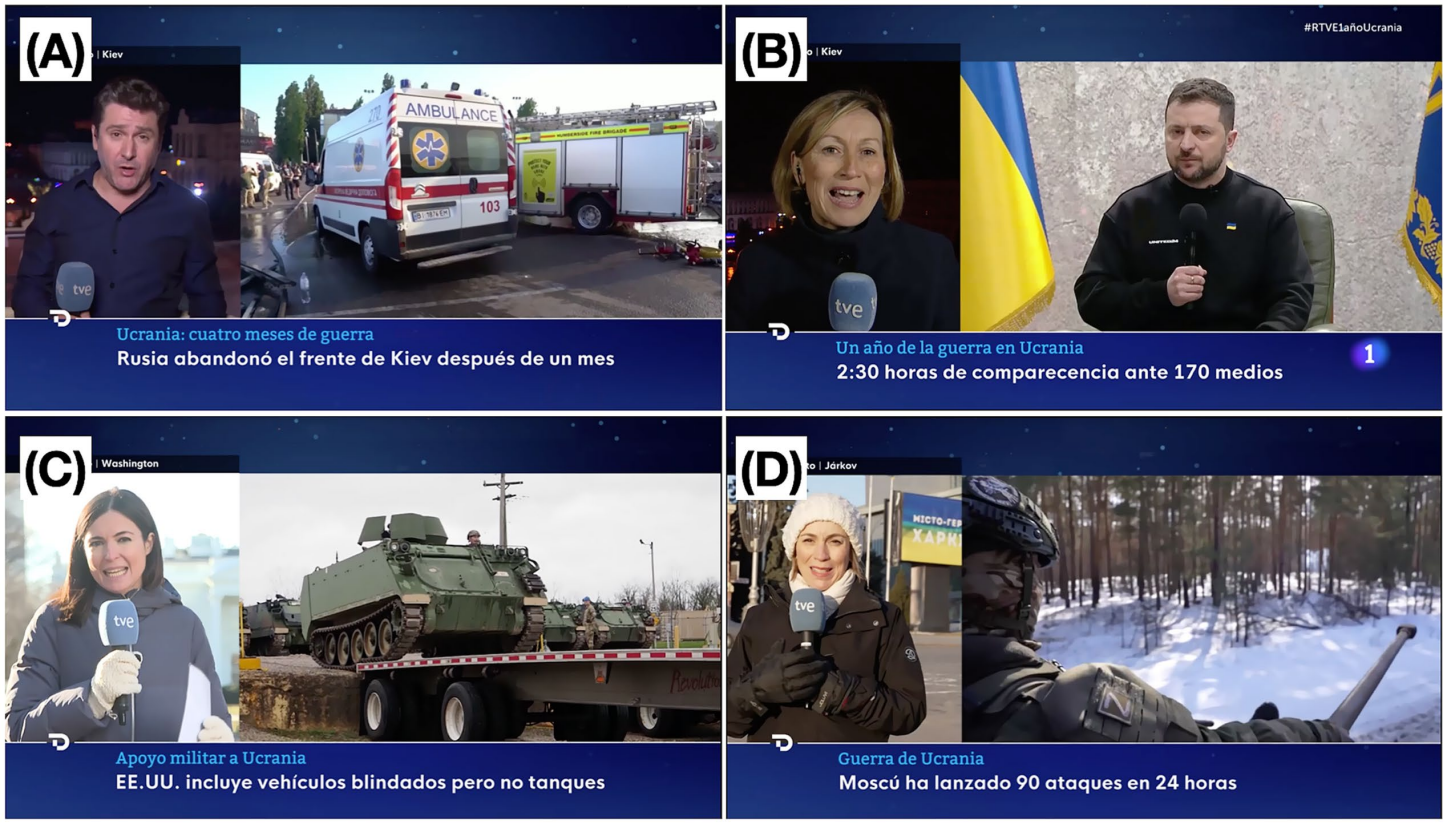
Sample of some of the images presented in the study. **(A)** A journalist is reporting from the trenches. **(B)** A journalist if reporting from a war-torn area. **(C)** A journalist is reporting in front of some tanks. **(D)** A journalist is reporting in from of soldiers. Still image reproduced with permission from RTVE.

We created a sample of 30 images of journalists reporting on the war in Ukraine. All images were captured from the news broadcast of Channel 1 of Radio Televisión Española, RTVE (Spanish Public Radio Television) (see Figure 1). A total of 30 still images were presented in randomized order, each displayed for 8 s. All images were drawn from Spanish TV news coverage of the war in Ukraine and featured journalists reporting from various conflict-related contexts. The scenes included locations such as trenches, bombed towns, rubble-filled streets, military zones, and migration points. Several images depicted interactions with civilians or soldiers, while others showed emergency responders, destroyed infrastructure, or military equipment. Notably, some frames included emotionally intense content, such as deceased bodies, images of President Zelensky or President Putin, and scenes of humanitarian distress (for more detail of the images, see Supplementary material). The still frames used as stimuli were captured from RTVE PLAY, the official online platform of Spain’s public television broadcaster. These online versions reproduce the same visual content as the original linear TV broadcasts, with no differences in framing or composition.

### 3.3 Setup and protocol

We used a remote eye-tracking device (GP3 HD, Gazepoint) connected to a monitor for visual stimulus presentation and to a computer for data management. Calibration of participants’ eyes was performed using Gazepoint Control software, while data acquisition was conducted with Gazepoint Analysis, both running on a Windows operating system. The eye-tracking experiment was conducted using a 27-inch HP monitor with resolution of 1920 × 1,080 pixels. Participants were seated approximately 60 cm from a screen with a 16:9 aspect ratio, resulting in a field of view (FOV) of approximately 53° horizontally and 31° vertically.

**Figure 2:**
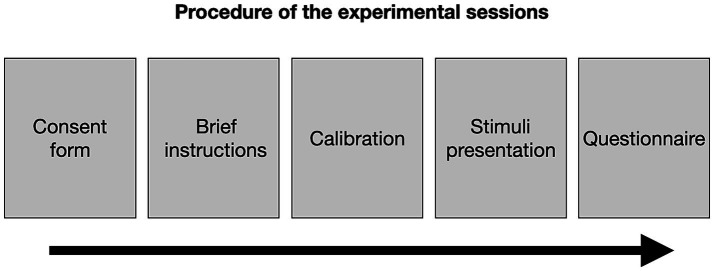
First, participants signed an informed consent form and received brief instructions explaining that they would view a series of images on the screen and subsequently complete a questionnaire related to those images. Next, eye-tracking calibration was performed using the manufacturer’s software to ensure accurate data collection. Once calibration was complete, the visual stimuli were presented in a randomized order. After viewing the images, participants completed a paper-based questionnaire on site.

Participants first entered the experiment room, where they were informed about the study and signed a consent form (Figure 2). They were then seated in front of the screen, which was connected to a laptop (Windows OS) used for presenting the images on the external screen. Before the image presentation, their gaze was calibrated using Gazepoint Control to ensure accurate eye-tracking data. Once calibration was complete, a total of 30 images were displayed in a randomized order, each shown for 8 s, using Gazepoint Analysis. Throughout the experiment, an eye-tracking device recorded their gaze patterns. After viewing all the images, participants completed a paper-based questionnaire on site, which they filled out manually. The questionnaire assessed their responses and perceptions, including emotional evaluations, and collected basic demographic and additional information (Supplementary material). Note that no facial detection software was used for emotion detection in this experiment.

### 3.4 Data acquisition and analysis

Using an eye-tracking device, we recorded subjects’ ocular biometric data. Our goal was to analyze their gaze patterns to determine which area of interest (AOI) attracted the most visual attention. To assess this, we analyzed the time that viewers spent on each AOI, the number of fixations they made, and the revisits. After the presentation of the images, we also administered a questionnaire to participants to evaluate their memory of the presented images and their emotional responses to war reporting. The complete questionnaire is available in the Supplementary material. To address the emotional responses, we used Paul Ekman’s six emotions (Ekman, 2003): sadness, joy, anger, fear, disgust, and surprise, employing a 1–5 Likert scale. It is important to note that Ekman’s framework was used solely as a reference for participants’ self-reported emotions, not for objective or physiological emotion analysis as originally proposed in his model.

Once the data were collected, we organized the stimuli into clusters based on relevant content characteristics that we aimed to compare (e.g., images depicting deceased individuals versus those without). For each comparison, we tested the normality of the data using the Shapiro–Wilk test. Depending on the outcome, we conducted either parametric or nonparametric repeated-measures analyses of variance, as well as *t*-tests, to assess differences between conditions. A significance level of *p* < 0.05 was used as the threshold for statistical significance.

## 4 Results

### 4.1 Visual attention to elements in war reporting

We compared how viewers distributed their gaze when three sections were present in the image: the section containing the journalist, the section of the picture presenting war images, and the section showing informative text (including the name of the journalist, the headline, or the location from which it was reported). We obtained significant differences (*X^2^* (2, *N* = 49) = 47.510, *p* < 0.001, Friedman repeated-measures ANOVA on ranks), with the longest gaze duration (mean±SD) on war images (3.213 ± 0.471), followed by the text section (2.146 ± 0.276), and finally, the journalists’ image (1.159 ± 0.481).

**Figure 3:**
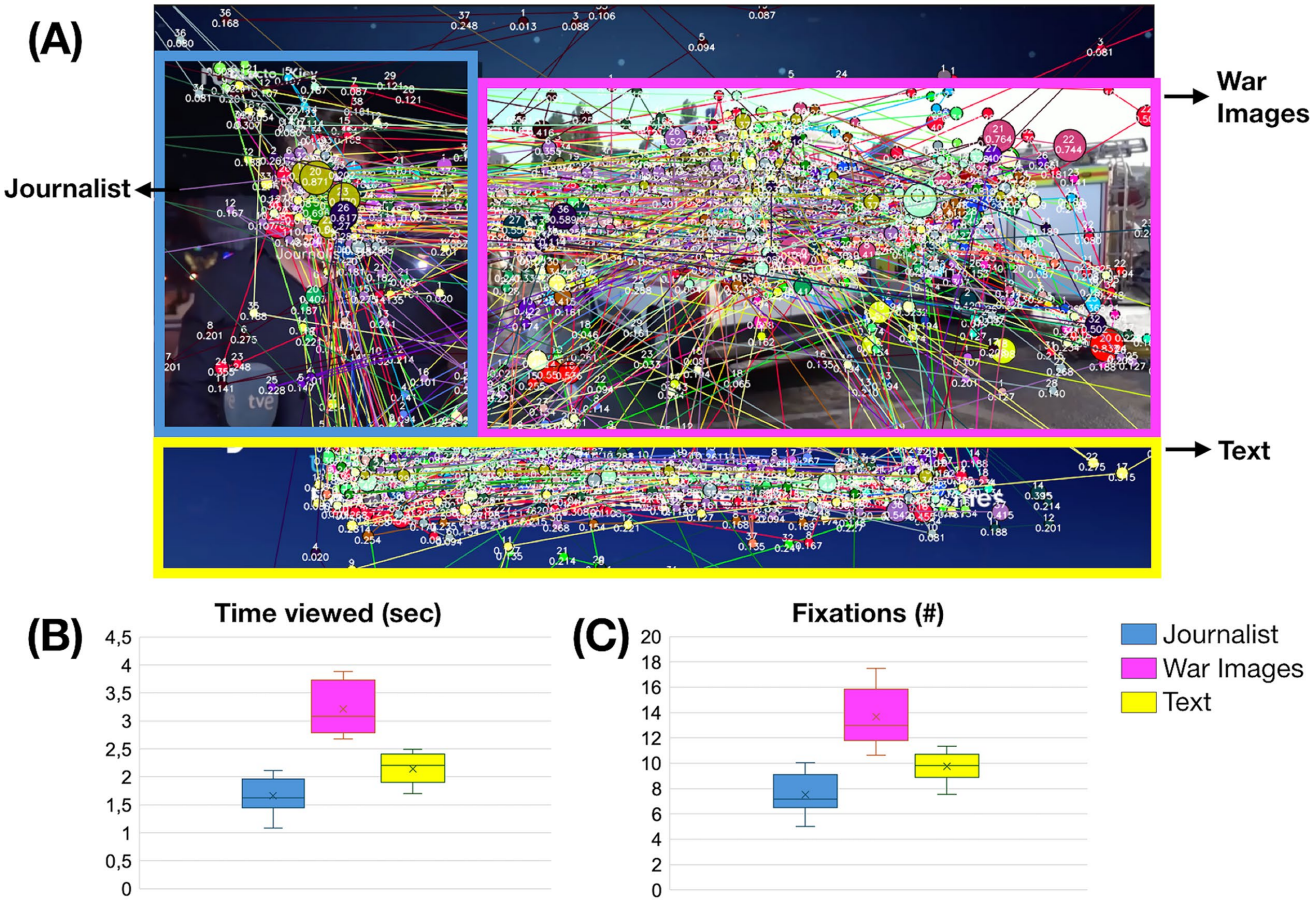
Distribution of gaze across the three conditions within the images: journalists, war images, and texts. Each line represents a participant’s gaze, with dots indicating fixations. **(A)** Example of a standard image with the three sections marked. **(B)** Average (mean±SD) viewing time (in seconds) across the three conditions. **(C)** Average [mean(±SD)] number of fixations across the three conditions.

We also analyzed the fixations in this regard and also obtained significant differences (*X^2^* (2, *N* = 49) = 46.082, *p* < 0.001, Friedman repeated-measures ANOVA on ranks), with the highest number of fixations for the war images (13.686 ± 2.397), followed by the text sections (9.746 ± 1.205), and the journalists’ images (7.509 ± 1.517) (see Figure 3).

### 4.2 Impact of deceased individuals on visual perception

**Figure 4:**
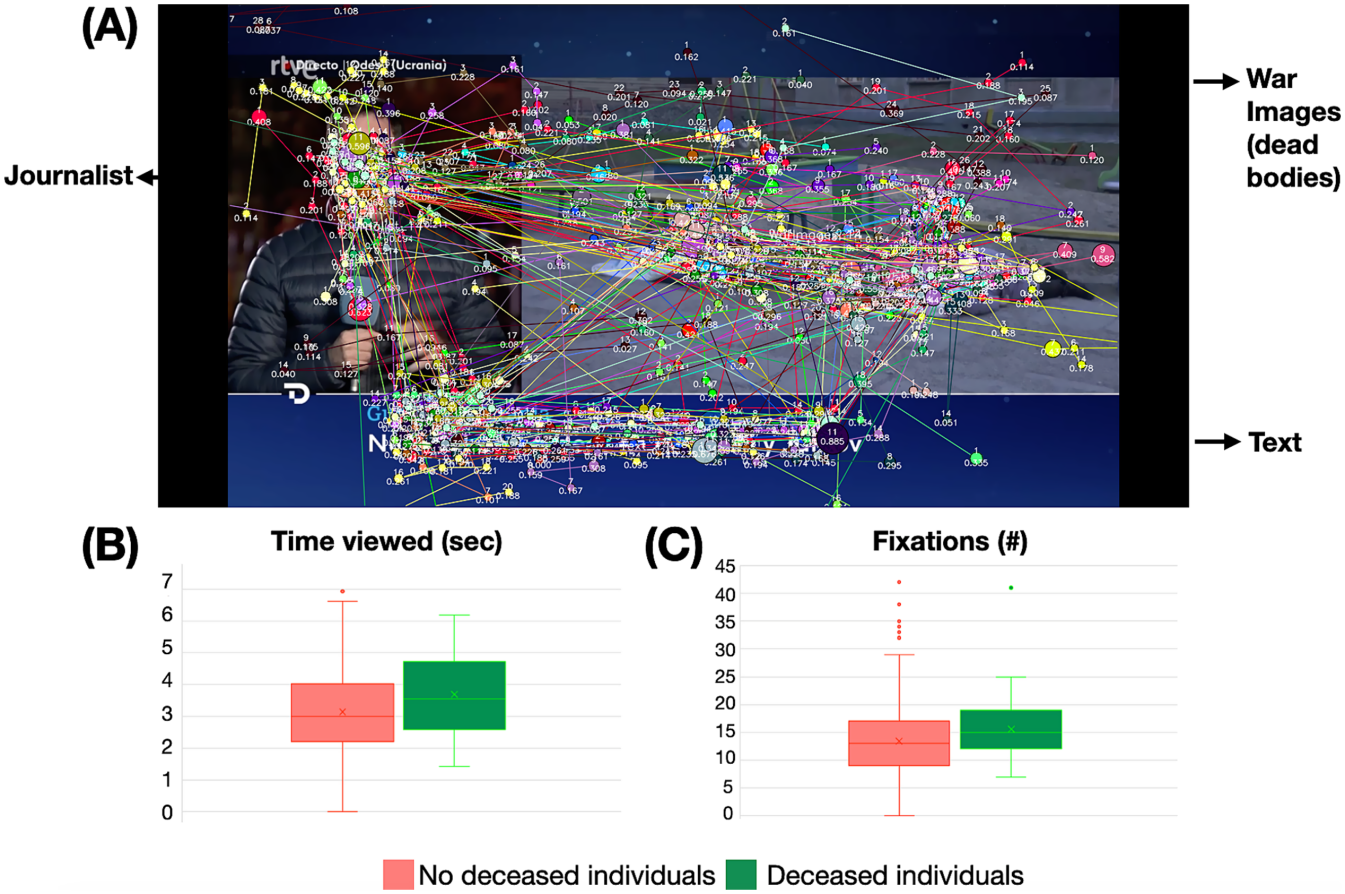
Distribution of gaze in war images with deceased individuals. **(A)** Participants’ gaze with war images including deceased individuals. **(B)** Average (mean±SD) viewing time (in seconds) across the war images including deceased individuals and without deceased individuals. **(C)** Average [mean(±SD)] number of fixations across the war images including deceased individuals and without deceased individuals.

We compared the time that viewers looked at war images presenting deceased individuals versus the time they looked at war images without deceased people. We obtained significant differences (*t*_(48)_ = −3.154, *p* = 0.003, *t*-test) with a higher time (mean±SD) dedicated to images with deceased individuals on them (3.689 ± 1.343) than without them (3.147 ± 0.824). When considering fixations, we also found a higher number in images with deceased individuals (15.592 ± 6.072) than in other war images (13.420 ± 4.115), againt obtaining a significant difference (*t*_(48)_ = −3.696, *p* < 0.001, *t*-test) (see Figure 4).

We found that, within images containing deceased individuals, journalists, and textual elements, the number of revisits was highest for the deceased individuals (2.061 ± 1.298) followed by the journalists (1.837 ± 1.675), and lowest for the textual content (0.837 ± 1.124), with a significant difference (*X^2^* (2, *N* = 49) = 25.762, *p* < 0.001, Friedman repeated-measures ANOVA on ranks). However, a comparison between the revisits to images depicting deceased individuals and those illustrating other war-related scenes without fatalities revealed that the former (2.061 ± 1.298) elicited significantly lower revisit rates than the latter (2.596 ± 1.759) (*t*_(48)_ = 3.067, *p* = 0.004, *t*-test).

### 4.3 Visual attention to leaders of opposing war factions: Zelenski vs. Putin

**Figure 5:**
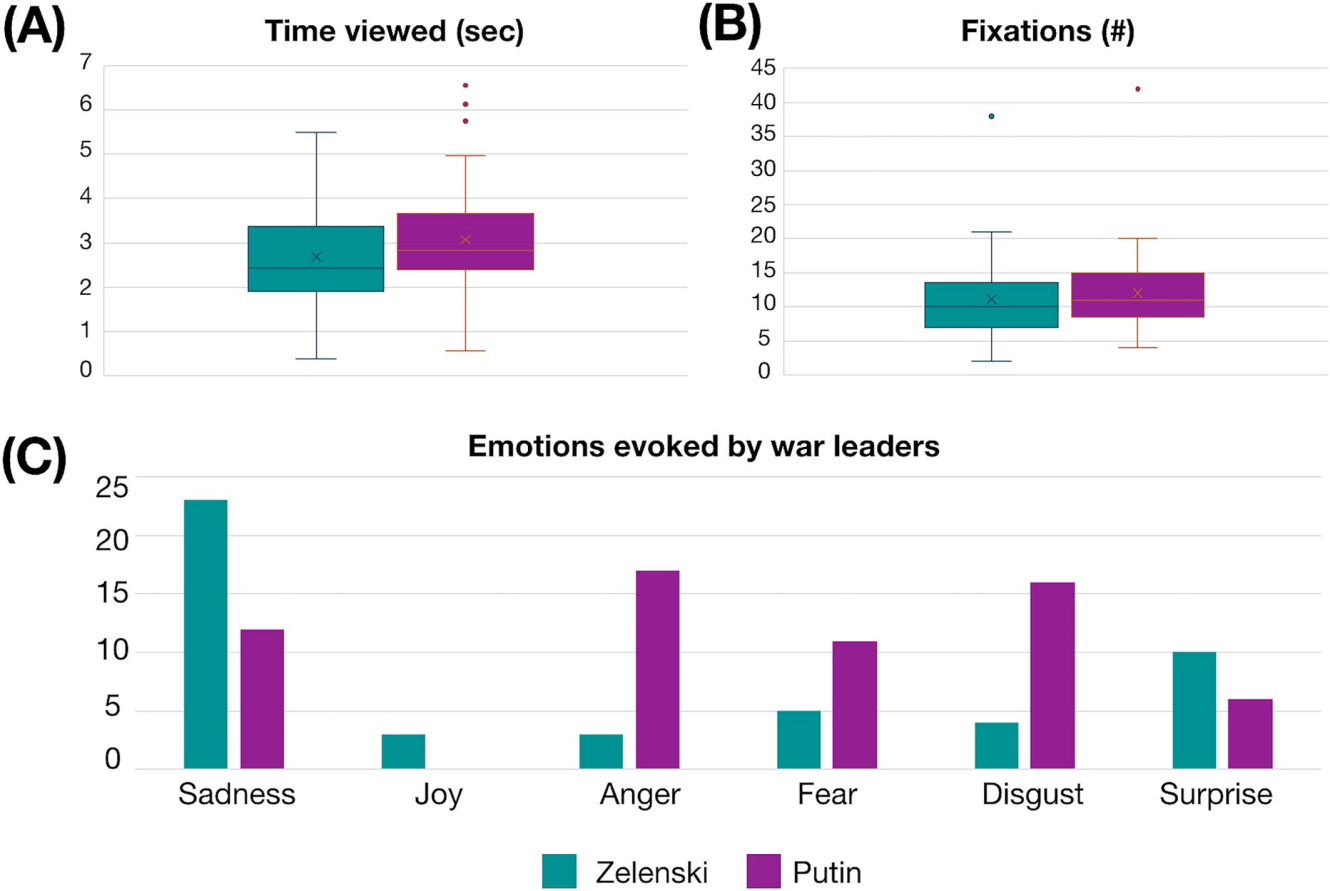
Visual attention and emotional responses to war leaders. **(A)** Mean viewing time (±SD) dedicated to war leaders’ images. **(B)** Mean number of fixations (±SD) on war leaders’ images. **(C)** Emotional responses evoked by war leaders’ images.

We were interested in evaluating potential differences in visual gaze toward the leaders of the war factions, Volodymyr Zelenski and Vladimir Putin. In terms of the time dedicated to the images of both leaders, longer duration was observed in Putin (in sec) (mean±SD) (3.069 ± 1.197) than Zelenski (2.681 ± 1.235) (in sec), which reached statistical significance (*t*_(48)_ = −2.302, *p* = 0.026, *t*-test). In terms of fixations, Putin also provoked a higher number of them (12.020 ± 6.012) than Zelenski (11.082 ± 5.951), but with no significant difference (*t*_(48)_ = −1.563, *p* = 0.125, *t*-test) (see Figures 5A,B).

### 4.4 Emotional responses to war reporting

To assess emotional responses to war reporting, first, we asked participants if, at the beginning of the war in Ukraine, the images were more impactful on a 1–5 Likert scale, ranging from 1 (strongly disagree) to 5 (strongly agree); the results were 1 (4.1%), 2 (8.2%), 3 (24.5%), 4 (28.6%) and 5 (34.7%). We then asked about the emotions that best described war information (see Table 1). We found that most participants felt sadness when exposed to war coverage and images of Zelensky, Putin primarily elicited feelings of disgust, and watching both Ukrainian and Russian citizens triggered feelings of sadness (see Figure 5C).

**Table 1:** Emotions that war images provoked to participants.

The emotion(s) that best describes what I feel when I see on TV…	Sadness [*N* (%)]	Joy [*N* (%)]	Anger [*N* (%)]	Fear [*N* (%)]	Disgust [*N* (%)]	Surprise [*N* (%)]
… war information is…	37 (75.5%)	0 (0%)	16 (32.7%)	16 (32.7%)	7 (14.3%)	4 (8.2%)
… Zelenski is…	23 (52.3%)	3 (6.8%)	3 (6.8%)	5 (11.4%)	4 (9.1%)	10 (22.7%)
… Putin is…	12 (24.5%)	0 (0%)	17 (34.7%)	11 (22.4%)	16 (32.7%)	6 (12.2%)
… Ukranian citizens…	45 (91.8%)	1 (2%)	6 (12.2%)	3 (6.1%)	0 (0%)	3 (6.1%)
… Russian citizens…	36 (75%)	1 (2.1%)	6 (12.5%)	3 (6.3%)	2 (4.2%)	12 (25%)

### 4.5 Audience recall of war image content

To assess participants’ memory of the images presented, we asked them (1) whether all the journalists visibly wore press identification, which 46.9% answered correctly; (2) whether all the journalists were in a war zone, which 57.1% answered correctly; (3) whether there were more male or female journalists in the images, which 83.7% answered correctly; (4) what color the journalists’ microphones were, which 91.8% answered correctly; (5) and whether the journalists in the war zone were wearing helmets, which 71.4% answered correctly. Computed jointly, 64.2% of the answers were correct whereas 35.8% were incorrect.

A more challenging task was introduced to assess participants’ memory. In this task, they had to identify the type of content presented in the images by selecting from a list of options. The list comprised 21 choices, including 16 that accurately reflected the content shown in the war images and 5 that depicted content that was not present in the images. No participant answered all the items correctly. For the options representing content that had indeed appeared in the war images, 73.3% of the responses were correct whereas 26.6% were incorrect, indicating that participants failed to recall certain elements that had been presented. This suggests that individuals did not fully remember all the content shown in the 30 images. Regarding the options describing content that had not been present in the images, 93.9% of the responses were correct whereas 6.1% were incorrect, suggesting that participants predominantly did not fabricate content that had not been shown.

## 5 General discussion

This study investigated the visual perception of still frames depicting war, extracted from Spanish television news broadcasts. The stimuli in this experiment were images presented within three distinct windows on a screen display (Figures 1, 3): a window showing the journalist speaking, a window displaying war-related footage, and a banner containing captions and additional information. These visual structures are now common in contemporary Spanish news broadcasts, as well as in sports and entertainment programs. The longest gaze durations were observed for the window containing the pre-recorded footage, despite the fact that these images are often repeated to visually support the journalist’s live commentary. There is a scarcity of studies focusing on viewer attention in multi-window television news formats (Rodrigues et al., 2012), which paradoxically tend to emphasize the role of the news presenter over that of the accompanying images and windows.

More research has been conducted on attention and observation across multiple simultaneous screens (Vatavu and Mancas, 2014; Jay et al., 2013), highlighting delays in comprehension when viewers begin processing one type of information over another. Moreover, multi-window news formats share several similarities with the way information and attention are distributed on web pages (Peker et al., 2021; Lapa, 2007; Lee and Ahn, 2012). Studies in this area consistently show that images capture viewer attention more effectively than other elements, a pattern also observed in the present experiments. Recent research has also nuanced and qualified the importance of banner placement in web contexts, suggesting reduced comprehension and shorter viewing times on the part of web users (Simonetti and Bigne, 2024). Previous studies conducted by our research team have shown that the cognitive load imposed by images—specifically in the context of scientific outreach posters—results in lower retention and retrieval of information compared with when the same content is presented in text-only format. Thus, an image is not always worth a thousand words (Martín-Pascual, 2024).

The mechanisms underlying memory function, particularly when emotional factors are involved, are complex and multifaceted. These processes may be influenced by the mode of information presentation (Steinmetz and Kensinger, 2013), as in this case, where images from news reports on conflict and war were used. Research employing eye-tracking techniques has demonstrated that many consolidated memories correspond with oculomotor behavior—specifically, with fixation locations and durations (Ryan and Shen, 2020). A higher number of fixations on deceased individuals (Figure 3) is to be expected; however, the longer viewing times observed are particularly noteworthy, as they may indicate the selective engagement of memory processes. Other research groups have focused on violence and its associated instruments. A well-known phenomenon is the ‘weapon focus’ effect, whereby increased fixations on firearms result in impaired recall of other image details (Loftus et al., 1987).

Equally significant is the role of gaze revisits as a cognitive mechanism supporting visual memory representation. The saccades preceding these revisits are thought to be driven initially by the dorsal attentional network, supported by working memory functions in the visual cortex and scene localization (Nikolaev et al., 2024). At this point, the hippocampal memory system is recruited, as it involves higher-order conscious understanding of what is being observed (Nikolaev et al., 2025). It is increasingly evident that revisits and recurrent fixations function as strategic components in memory management, with each revisit contributing to long-term memory consolidation. This may explain why certain areas of interest (AOIs) within an image are re-examined—to facilitate the transition from short-term to long-term memory. In this experiment, for images depicting death, the average number of revisits to the area containing deceased individuals was higher compared with other areas. However, when comparing war-related footage with and without deceased individuals, the absence of deceased individuals was associated with a higher number of revisits.

Regarding cultural bias in the emotional responses elicited by the opposing leaders in the conflict and the selection of stimuli in this study featuring images of the war’s effects on Ukrainian territory, the findings highlight the importance of memory and the influence of media representations. Prior to the full-scale war, public awareness of Volodymyr Zelensky and the events following the Euromaidan movement in March 2014—culminating in the occupation of Donbas—was relatively limited and had less impact in the West compared with the 2022 invasion. The latter, framed by the Kremlin as a purported ‘denazification’ of Ukraine (Shekhovtsov, 2015), brought greater international attention, partly due to the more prominent media profile of Vladimir Putin, who was significantly better known than Zelensky at the time. There is a marked difference in public perceptions between those who desire peace and the trajectory of the conflict’s developments (Daniels and Polegkyi, 2025). Moreover, pro-Russian arguments have also been disseminated differently across various media outlets, often carrying emotional valences that differ significantly from those observed in the participants of this study. Misinformation plays a critical role in shaping public opinion and emotional responses to the conflict (Bliuc and Muntele‐Hendreș, 2025). It would be of great interest to present these stimuli in other cultural contexts and in countries within the Russian sphere of influence.

Finally, regarding the recall and memory of participants viewing images of conflict and war, participants demonstrated incorrect recollection or knowledge in nearly one-third of the cases. However, in surveys that included options that were not present in the images, only 6% of participants reported remembering details that were not part of the stimuli. This low incidence of false memory generation is noteworthy. It may reflect distinct patterns of behavior in the representation of images or text, as evidenced by the attention shown in the fixations (Karaca et al., 2025). Alternatively, it may suggest that, within the set of stimuli presented, similar images were erroneously retained owing to their similarity or, as indicated by other research, owing to differences in the way images are explored, which can be attributed to exploration patterns that vary with age or the observer’s training (Danilov et al., 2019). A more detailed future study on memory, considering age and professional background, would be beneficial.

## 6 Limitations

In this study, we wanted to pay attention to the gaze without audio contamination; for this reason, we used static images instead of video. However, this is one limitation of the study, since viewers of broadcasts watch news in video format. This choice reduces the ecological validity of our findings, as it does not fully replicate the multimodal nature of real news consumption, where movement, audio, and narrative structure significantly influence viewers’ attention and emotional responses. Future research could incorporate dynamic audiovisual content to better simulate real-world media exposure. A second limitation of the study is that the sample was not very large. Including a larger number of participants would have strengthened the results of this study. A third limitation of the present study is that the sample was not as gender balanced as desired. Another limitation is related to the approach to emotions. While this study used Paul Ekman’s six basic emotions (Ekman, 2003) as a reference framework for participants’ emotional responses, we recognize the limitations of this categorical model in capturing the nuances of emotional experience, particularly in relation to physiological data, e.g., obtained by eye-tracking. Other models focusing on valence and arousal may offer a more integrative approach for combining emotion measurement with psychophysiological data (Müller et al., 2012). The use of facial detection software would have enhanced the validity of applying Ekman’s system in this study. Another limitation of this study is the potential influence of ongoing media coverage on participants’ perceptions and responses, as data collection took place during a highly publicized period. However, the study began over a year after the initial Ukraine invasion, meaning participants had already been exposed to established media narratives. Another important limitation of this study concerns the ethical procedures related to the use of sensitive visual stimuli, specifically images depicting deceased individuals. No formal debriefing process was implemented, nor were participants provided with contact information for professional psychological support. Given the potentially distressing nature of the images—and our findings that many participants revisited particularly sensitive content—this lack of follow-up support is a significant limitation. Future research should ensure comprehensive debriefing protocols and offer appropriate resources to participants to address any emotional distress arising from exposure to such material. A further limitation of this study is the absence of self-perceived arousal measurements for the different categories of images presented. We did not collect specific arousal ratings for each image or category, which could have provided a more nuanced understanding of how emotional intensity correlates with visual attention. Future research should incorporate arousal assessments alongside eye tracking to better capture the interplay between affective arousal and gaze behavior, particularly when analyzing negative or emotionally charged media content.

## 7 Conclusion

Eye tracking is a valuable tool that reflects oculomotor behavior in attention processes and memory consolidation. This study aimed to explore how war images are visually perceived in broadcast news. Specifically, we sought to understand how visual attention is distributed across different elements of war reporting when journalists, war images, and explanatory text are presented simultaneously. Our findings revealed that viewers paid more attention to the war images and explanatory text than to the journalists themselves. This suggests that the personal focus often employed by many media outlets, which places emphasis on the journalist, may be less relevant to viewers. Instead, they tend to focus more on the visual information than on the journalist’s presence. Future research should examine the greater cognitive load that images may impose compared with text or a static presenter or journalist. Additionally, it is important to investigate whether moving images constitute an additional attentional factor. Motion may elicit an innate response geared toward engaging with reality, facilitating motion detection and consequently activating deeper memory resources, as will be discussed below.

The news industry tends to self-censor and avoid displaying images of the dead (Fishman, 2017), so here we also wondered whether an image depicting deceased individuals would provoke a different visual perception compared with other war images. Indeed, we found that images with deceased people increased both the time viewers spent looking at them and the number of visual fixations, reflecting long-term memory construction processes, despite the decrease of revisits.

On the other hand, we asked whether the leaders of opposing war factions, such as Zelensky and Putin, had generated distinct visual attention patterns in the audience, and we wondered about the emotions that both leaders would evoke in viewers and whether those emotions would differ. We found that Putin received a higher quantity of attention, with more time spent on him and a higher number of fixations compared with Zelensky, as well as a higher level of negative emotions such as anger, fear, and disgust. Given that this study was conducted in a Western context (Barcelona, Spain), the findings may reflect a bias related to the participant sample. A comparison with participants from Russian territory would be valuable for complementing and broadening the scope of this research.

We explored whether participants were able to recall the content of the war images as presented in the media. Given our use of static screenshots—rather than full video segments—this assessment focused on memory of isolated visual content rather than multimodal narratives. While no participant answered all the items correctly, most responses accurately identified content present in the images, though some details were overlooked. Importantly, when asked about elements not depicted, most participants correctly avoided fabricating information.

Finally, an important methodological consideration for future studies is to directly compare complex images that simultaneously present multiple competing elements—such as a journalist, on-screen text, and war imagery—versus simpler images that isolate each element individually. Such a contrast would allow researchers to better understand how attentional resources are allocated when multiple visual stimuli vie for attention, and whether certain elements dominate gaze behavior depending on their emotional or informational salience. We would expect that complex images generate more divided or selective attention patterns, with potential prioritization of emotionally salient war images or faces of political figures, while simpler images might elicit more focused gaze on the single presented object. Exploring these differences could further clarify the dynamics of visual attention in broadcast news contexts.

## Data Availability

The raw data supporting the conclusions of this article will be made available by the authors, without undue reservation.
