# Supplementary material for: Visual perception of war images in Spanish TV news: an eye-tracking study using still frames
Source: Front Neurosci. 2025 Jul 9;19:1612487. doi: 10.3389/fnins.2025.1612487 (PMC12283630; doi:10.3389/fnins.2025.1612487)
Supplement: Supplementary file 1 [file Data_Sheet_1.docx]

Supplementary Material

# Supplementary Data

The 30 images included the following content:

1. A journalist was reporting from the trenches.

2. A journalist was reporting in front of some soldiers.

3. A journalist was reporting from a war-ravaged town, next to armored vehicles.

4. A journalist was reporting while images of a devastated area and fire trucks extinguishing a fire were shown.

5. A journalist was reporting while an image of an ambulance and a fire truck was displayed.

6. A journalist was reporting while firefighters were shown in a rubble-filled area.

7. A journalist was reporting from the trenches alongside a soldier.

8. A journalist was reporting in front of a hospital.

9. A journalist was reporting in a war-ravaged area full of debris.

10. A journalist was reporting in another combat-ravaged area filled with debris.

11. A journalist was interviewing a resident of an affected area.

12. A journalist was interviewing a female resident of an affected area.

13. A journalist was reporting while images of a fire in a populated area were shown.

14. A journalist was reporting while two bodies covered with sheets were seen on the ground.

15. A journalist was reporting while images of a military officer briefing from the Pentagon in Washington were shown.

16. A journalist was reporting from a war zone while in the background, two elderly women were seen placing bags of food on a bicycle.

17. A journalist was interviewing two elderly residents carrying plastic bags with food in one of the towns affected by the war.

18. A journalist was reporting in front of a fire truck with several firefighters on it, in a destroyed area.

19. A journalist was reporting in front of several tanks.

20. A journalist was reporting from the top of a residential building destroyed by bombs.

21. A journalist was reporting from a train station where dozens of people were migrating.

22. A journalist was reporting from a building destroyed by bombs.

23. A journalist was reporting in front of a house with its roof and windows shattered by bombs.

24. A journalist was reporting from an area full of rubble.

25. A journalist was reporting in front of two houses destroyed by bombs.

26. A journalist was reporting while a soldier was seen from behind in a snowy forest.

27. A journalist was reporting from a bridge destroyed by bombs.

28. A journalist was reporting while an image of Zelensky was shown.

29. A journalist was reporting while an image of Putin was shown.

30. A journalist was reporting while images of various tanks with soldiers were displayed

# Supplementary Data

Questionnaire (English translation):

| **Question** | **Open-ended or closed-ended items** |
| --- | --- |
| Gender | Female. Male. Prefer not to say |
| Age |  |
| Profession |  |
| Are you a journalis? | Yes. No |
| Have you covered any armed conflict on the ground? | Yes. No |
| Have you reported on the war? | Yes. No |
| Hand dominance | Left-handed. Right-handed. Ambidextrous |
| About your vision | I completed the test wearing glasses.  I completed the test wearing contact lenses. I’ve had laser eye surgery for myopia. I’ve had other eye surgery. Other: |
| Name any journalists you remember from the images |  |
| Were all the journalists clearly wearing visible press identification? | Yes. No |
| Were all the journalists in war zones? | Yes. No |
| Were there more male or female journalists in the images you saw? | Male. Female |
| What color(s) were the microphones the journalists were using, and what logo(s) did they display? |  |
| The photographs showed images of the war in Ukraine. Please check all the elements you remember seeing | A trench. A crane. Weapons. A bicycle. A hospital. A supermarket. Corpses. A fire. A pregnant woman. Putin. People crying. An ambulance. Biden. A child. Kyiv. Destroyed buildings. Soldiers. Firefighters. Civilians from conflict zones. Tanks. Zelensky |
| Did the journalists in the war zone wear helmets? | All. None. Some |
| I would go as a war correspondent | Strongly disagree 1 2 3 4 5 Strongly agree |
| War correspondents take too many risks to cover the news | Strongly disagree 1 2 3 4 5 Strongly agree |
| War reporting should show the reality of war without censorship, including images of dead bodies | Strongly disagree 1 2 3 4 5 Strongly agree |
| War reporting should preferably be done from the “good” side, that is, the victim’s side | Strongly disagree 1 2 3 4 5 Strongly agree |
| Journalists who go to war should show their faces on the news to give more value to their work | Strongly disagree 1 2 3 4 5 Strongly agree |
| At the beginning of the war in Ukraine, the images affected me more | Strongly disagree 1 2 3 4 5 Strongly agree |
| The emotion that best describes how I feel when I see war coverage is: | Sadness. Joy. Anger. Fear. Disgust. Surprise |
| The emotion that best describes how I feel when I see Zelensky is: | Sadness. Joy. Anger. Fear. Disgust. Surprise |
| The emotion that best describes how I feel when I see Putin is: | Sadness. Joy. Anger. Fear. Disgust. Surprise |
| The emotion that best describes how I feel when I see Ukrainian citizens on the news is: | Sadness. Joy. Anger. Fear. Disgust. Surprise |
| The emotion that best describes how I feel when I see Russian citizens on the news is: | Sadness. Joy. Anger. Fear. Disgust. Surprise |
| Thank you very much for your answers. Your participation is very valuable. If you would like to share any comments or opinions: |  |
